# Supplementary material for: A UMLS-based spell checker for natural language processing in vaccine safety
Source: BMC Med Inform Decis Mak. 2007 Feb 12;7:3. doi: 10.1186/1472-6947-7-3 (PMC1805499; doi:10.1186/1472-6947-7-3)
Supplement: Additional file 13 — RAP application directory. Sets up directory for RAP (RDF application for PHP) source code [file 1472-6947-7-3-S13.gz › rap/doc/phpdoc/__filesource/fsource_default__vocabularyDC_RES.php.html]

xml version="1.0" encoding="iso-8859-1"?


File Source for DC\_RES.php


# Source for file DC\_RES.php

Documentation is available at DC\_RES.php

```
1. <?PHP
2. // ----------------------------------------------------------------------------------
3. // Dublin Core Vocabulary (ResResource)
4. // ----------------------------------------------------------------------------------
5. // Version                   : 0.9
6. // Authors                   : Chris Bizer (chris@bizer.de)
7. //
8. // Description               : Wrapper, defining resources for all terms of the Dublin
9. //                               Core Vocabulary. For details about DC see: http://dublincore.org/
10. //                                Using the wrapper allows you to define all aspects of
11. //                             the vocabulary in one spot, simplifing implementation and
12. //                             maintainence.
13. //
14. // ----------------------------------------------------------------------------------
15. // <BR><BR>History:<UL>
16. // <LI>07-26-2004                 : Update for latest DC recs; changed suffixed globals to
17. //                                  array items.(auer@informatik.uni-leipzig.de)</LI>
18. // <LI>02-21-2003                 : Initial version (evan@wikitravel.org)</LI>
19. // ----------------------------------------------------------------------------------


27. class DC_RES{
28. // DC concepts

30. function CONTRIBUTOR()
31. {
32. return  new ResResource(DC_NS . 'contributor');

34. }

36. function COVERAGE()
37. {
38. return  new ResResource(DC_NS . 'coverage');

40. }

42. function CREATOR()
43. {
44. return  new ResResource(DC_NS . 'creator');

46. }

48. function DATE()
49. {
50. return  new ResResource(DC_NS . 'date');

52. }

54. function DESCRIPTION()
55. {
56. return  new ResResource(DC_NS . 'description');

58. }

60. function FORMAT()
61. {
62. return  new ResResource(DC_NS . 'format');

64. }

66. function IDENTIFIER()
67. {
68. return  new ResResource(DC_NS . 'identifier');

70. }

72. function LANGUAGE()
73. {
74. return  new ResResource(DC_NS . 'language');

76. }

78. function PUBLISHER()
79. {
80. return  new ResResource(DC_NS . 'publisher');

82. }

84. function RIGHTS()
85. {
86. return  new ResResource(DC_NS . 'rights');

88. }

90. function SOURCE()
91. {
92. return  new ResResource(DC_NS . 'source');

94. }

96. function SUBJECT()
97. {
98. return  new ResResource(DC_NS . 'subject');

100. }

102. function TITLE()
103. {
104. return  new ResResource(DC_NS . 'title');

106. }

108. function TYPE()
109. {
110. return  new ResResource(DC_NS . 'type');
111. }


114. // Other Elements and Element Refinements

116. function ABSTRACT_()
117. {
118. return  new ResResource(DCTERM_NS . 'abstract');

120. }

122. function ACCESS_RIGHTS()
123. {
124. return  new ResResource(DCTERM_NS . 'accessRights');

126. }

128. function ALTERNATIVE()
129. {
130. return  new ResResource(DCTERM_NS . 'alternative');

132. }

134. function AUDIENCE()
135. {
136. return  new ResResource(DCTERM_NS . 'audience');

138. }

140. function AVAILABLE()
141. {
142. return  new ResResource(DCTERM_NS . 'available');

144. }

146. function BIBLIOGRAPHIC_CITATION()
147. {
148. return  new ResResource(DCTERM_NS . 'bibliographicCitation');

150. }

152. function CONFORMS_TO()
153. {
154. return  new ResResource(DCTERM_NS . 'conformsTo');

156. }

158. function CREATED()
159. {
160. return  new ResResource(DCTERM_NS . 'created');

162. }

164. function DATE_ACCEPTED()
165. {
166. return  new ResResource(DCTERM_NS . 'dateAccepted');

168. }

170. function DATE_COPYRIGHTED()
171. {
172. return  new ResResource(DCTERM_NS . 'dateCopyrighted');

174. }

176. function DATE_SUBMITTED()
177. {
178. return  new ResResource(DCTERM_NS . 'dateSubmitted');

180. }

182. function EDUCATION_LEVEL()
183. {
184. return  new ResResource(DCTERM_NS . 'educationLevel');

186. }

188. function EXTENT()
189. {
190. return  new ResResource(DCTERM_NS . 'extent');

192. }

194. function HAS_FORMAT()
195. {
196. return  new ResResource(DCTERM_NS . 'hasFormat');

198. }

200. function HAS_PART()
201. {
202. return  new ResResource(DCTERM_NS . 'hasPart');

204. }

206. function HAS_VERSION()
207. {
208. return  new ResResource(DCTERM_NS . 'hasVersion');

210. }

212. function IS_FORMAT_OF()
213. {
214. return  new ResResource(DCTERM_NS . 'isFormatOf');

216. }

218. function IS_PART_OF()
219. {
220. return  new ResResource(DCTERM_NS . 'isPartOf');

222. }

224. function IS_REFERENCED_BY()
225. {
226. return  new ResResource(DCTERM_NS . 'isReferencedBy');

228. }

230. function IS_REPLACED_BY()
231. {
232. return  new ResResource(DCTERM_NS . 'isReplacedBy');

234. }

236. function IS_REQUIRED_BY()
237. {
238. return  new ResResource(DCTERM_NS . 'isRequiredBy');

240. }

242. function ISSUED()
243. {
244. return  new ResResource(DCTERM_NS . 'issued');

246. }

248. function IS_VERSION_OF()
249. {
250. return  new ResResource(DCTERM_NS . 'isVersionOf');

252. }

254. function LICENSE()
255. {
256. return  new ResResource(DCTERM_NS . 'license');

258. }

260. function MEDIATOR()
261. {
262. return  new ResResource(DCTERM_NS . 'mediator');

264. }

266. function MEDIUM()
267. {
268. return  new ResResource(DCTERM_NS . 'medium');

270. }

272. function MODIFIED()
273. {
274. return  new ResResource(DCTERM_NS . 'modified');

276. }

278. function REFERENCES()
279. {
280. return  new ResResource(DCTERM_NS . 'references');

282. }

284. function REPLACES()
285. {
286. return  new ResResource(DCTERM_NS . 'replaces');

288. }

290. function REQUIRES()
291. {
292. return  new ResResource(DCTERM_NS . 'requires');

294. }

296. function RIGHTS_HOLDER()
297. {
298. return  new ResResource(DCTERM_NS . 'rightsHolder');

300. }

302. function SPATIAL()
303. {
304. return  new ResResource(DCTERM_NS . 'spatial');

306. }

308. function TABLE_OF_CONTENTS()
309. {
310. return  new ResResource(DCTERM_NS . 'tableOfContents');

312. }

314. function TEMPORAL()
315. {
316. return  new ResResource(DCTERM_NS . 'temporal');

318. }

320. function VALID()
321. {
322. return  new ResResource(DCTERM_NS . 'valid');

324. }


327. // Encoding schemes

329. function BOX()
330. {
331. return  new ResResource(DCTERM_NS . 'Box');

333. }

335. function DCMI_TYPE()
336. {
337. return  new ResResource(DCTERM_NS . 'DCMIType');

339. }

341. function IMT()
342. {
343. return  new ResResource(DCTERM_NS . 'IMT');

345. }

347. function ISO3166()
348. {
349. return  new ResResource(DCTERM_NS . 'ISO3166');

351. }

353. function ISO639_2()
354. {
355. return  new ResResource(DCTERM_NS . 'ISO639-2');

357. }

359. function LCC()
360. {
361. return  new ResResource(DCTERM_NS . 'LCC');

363. }

365. function LCSH()
366. {
367. return  new ResResource(DCTERM_NS . 'LCSH');

369. }

371. function MESH()
372. {
373. return  new ResResource(DCTERM_NS . 'MESH');

375. }

377. function PERIOD()
378. {
379. return  new ResResource(DCTERM_NS . 'Period');

381. }

383. function POINT()
384. {
385. return  new ResResource(DCTERM_NS . 'Point');

387. }

389. function RFC1766()
390. {
391. return  new ResResource(DCTERM_NS . 'RFC1766');

393. }

395. function RFC3066()
396. {
397. return  new ResResource(DCTERM_NS . 'RFC3066');

399. }

401. function TGN()
402. {
403. return  new ResResource(DCTERM_NS . 'TGN');

405. }

407. function UDC()
408. {
409. return  new ResResource(DCTERM_NS . 'UDC');

411. }

413. function URI()
414. {
415. return  new ResResource(DCTERM_NS . 'URI');

417. }

419. function W3CDTF()
420. {
421. return  new ResResource(DCTERM_NS . 'W3CDTF');

423. }


426. // DCMI Type Vocabulary

428. function COLLECTION()
429. {
430. return  new ResResource(DCMITYPE_NS . 'Collection');

432. }

434. function DATASET()
435. {
436. return  new ResResource(DCMITYPE_NS . 'Dataset');

438. }

440. function EVENT()
441. {
442. return  new ResResource(DCMITYPE_NS . 'Event');

444. }

446. function IMAGE()
447. {
448. return  new ResResource(DCMITYPE_NS . 'Image');

450. }

452. function INTERACTIVE_RESOURCE()
453. {
454. return  new ResResource(DCMITYPE_NS . 'Interactive_Resource');

456. }

458. function MOVINGIMAGE()
459. {
460. return  new ResResource(DCMITYPE_NS . 'Moving_Image');

462. }

464. function PHYSICALOBJECT()
465. {
466. return  new ResResource(DCMITYPE_NS . 'Physical_Object');

468. }

470. function SERVICE()
471. {
472. return  new ResResource(DCMITYPE_NS . 'Service');

474. }

476. function SOFTWARE()
477. {
478. return  new ResResource(DCMITYPE_NS . 'Software');

480. }

482. function SOUND()
483. {
484. return  new Resource(DCMITYPE_NS . 'Sound');

486. }

488. function STILLIMAGE()
489. {
490. return  new ResResource(DCMITYPE_NS . 'Still_Image');

492. }

494. function TEXT()
495. {
496. return  new ResResource(DCMITYPE_NS . 'Text');
497. }
498. }


502. ?>
```

Documentation generated on Fri, 17 Dec 2004 16:14:47 +0100 by phpDocumentor 1.3.0RC3
